# Application of galangin, an active component of *Alpinia officinarum* Hance (Zingiberaceae), for use in drug-eluting stents

**DOI:** 10.1038/s41598-017-08410-2

**Published:** 2017-08-15

**Authors:** Jung-Jin Lee, Ji-Hye Lee, Nam-Hui Yim, Joo-Hui Han, Jin Yeul Ma

**Affiliations:** 1 Korean Medicine (KM) Application Center, Korea Institute of Oriental Medicine, Daegu, 701-300 Republic of Korea; 2 Department of Pharmacology, Chungnam National University College of Pharmacy, Daejeon, 305-764 Republic of Korea

## Abstract

In clinical pathology, stent interposition is used to treat vascular disease but can lead to restenosis. Drug-eluting stents (DES) are most commonly used to suppress restenosis but can also have side effects. Therefore, we investigated the anti-proliferative effect and its possible target *in vitro* and *in vivo*. We found that *Alpinia officinarum* Hance (AO) extract efficiently inhibited VSMC proliferation by arresting the transition from the G_0_/G_1_ to the S phase via the up-regulation of p27^KIP1^ expression. Galangin (GA) was determined to be a significant component of this extract, with the same anti-proliferative activity as the raw extract. Immunoblotting and immunofluorescence staining showed that both the AO extract and GA targeted the up-regulation of p27^KIP1^ expression. Therefore, we next examined the effect of these compounds in a cuff-injured neointimal hyperplasia model *in vivo*. In this animal model, both the AO extract and GA completely suppressed the neointima formation, and this inhibitory effect was also demonstrated to target the up-regulation of p27^KIP1^, including the suppression of proliferating cell nuclear antigen expression. Our findings indicate that AO extract and GA have a potent anti-proliferative activity, targeting the up-regulation of p27 expression. Thus, GA may represent an alternative medicine for use in DES.

## Introduction

Vascular disease is caused by a number of factors, such as the inflammatory response to vascular injury, neointima hyperplasia by vascular smooth muscle cell (VSMC) proliferation^1^. Recently, vascular balloon dilatation involving stent interposition has been used to treat vascular disease in clinical pathology. However, this stenting can itself lead to restenosis accompanying neointima hyperplasia through the abnormal proliferation of VSMCs^2^. Commonly, neointima formation following surgical coronary intervention, particularly angioplasty, is caused by several cellular processes, among which abnormal VSMC proliferation is a principle contributing factor^3^. VSMC proliferation is initiated by cytokines and growth factors, such as platelet-derived growth factor (PDGF)-BB, which are secreted from macrophages. Early signal activation is caused by the growth factors binding to receptors, which induces cell cycle progression, including the expression of cyclins and cyclin-dependent kinases (CDKs)^4, 5^. The CDK/cyclin complexes are up-regulated as a transition point in the G_1_-S phase, resulting in the dissociation of transcription factor E2F, which forms a complex with and activates the retinoblastoma (Rb) protein and induces DNA synthesis via the expression of genes. These progressions in the cell cycle are regulated by CDK inhibitors (CKIs), such as p16, p21^WAF1/CIP1^, p27^KIP1^, and p53^6, 7^. Among these, p27^KIP1^ acts as a negative regulator that can arrest the cell cycle transition of the G_0_/G_1_ phase^8^ and is known to degrade on mitogenic stimulation^9^, and so is considered a therapeutic target in vascular disease^10, 11^.

The flavonoid 3,5,7-trihydroxyflavone (galangin; GA) has been reported to have a variety of biological activities, including antitumor, antimutagenic, antioxidative, bactericidal, and antifibrotic effects^12–15^. This compound is found in the rhizome of *Alpinia officinarum* Hance (AO), which has been used as a herbal medicine for colds, stomach aches, and swellings, or as a food additive for centuries in Asia. Moreover, GA has long been taken as a remedy for various symptoms, particularly in China^16^. Recent studies have demonstrated that GA has a novel function and specific activity in various disorders, such as allergic inflammation, atopic dermatitis-like skin lesions, and acute lung injury^17–19^. However, its effect on the cardiovascular system, including atherosclerosis and restenosis, is not yet known.

Therefore, in this study, we compared the function of AO extract and GA with that of paclitaxel and rapamycin (sirolimus), which is used to treat vascular disease in clinical pathology, on VSMC proliferation, and investigated their targets of action. We also verified their therapeutic effects in an animal model resulting from restenosis and atherosclerosis. Our findings suggest that GA has potential as an alternative drug to paclitaxel and rapamycin when treating vascular disorders.

## Results

### Action of the AO extract on VSMC proliferation and early signaling phosphorylation

**Figure 1:**
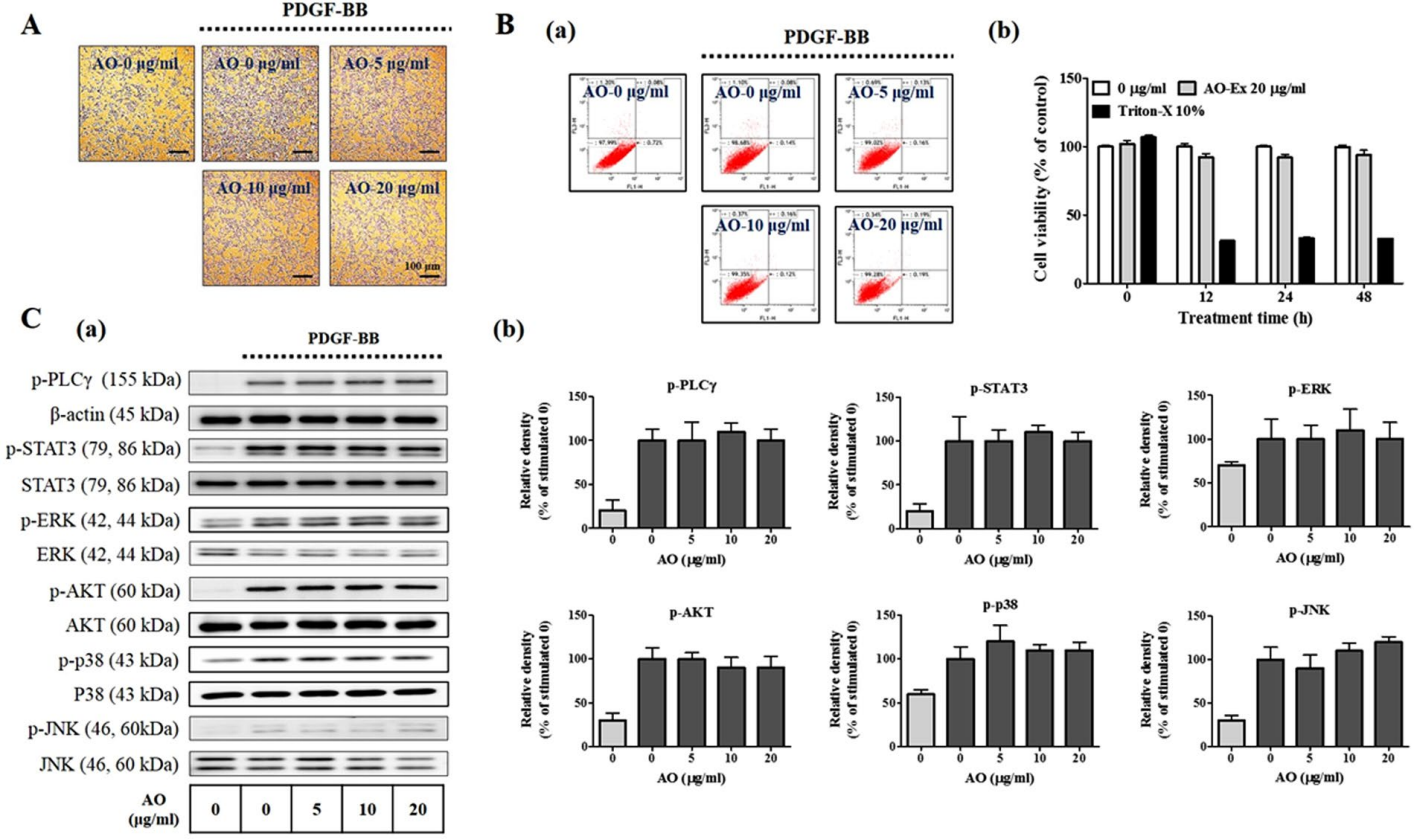
Effects of *Alpinia officinarum* Hance (AO) extract on the proliferation and early signaling transduction of vascular smooth muscle cells (VSMCs). Quiescent VSMCs cultured in serum-free medium were stimulated with 25 ng/mL platelet-derived growth factor (PDGF)-BB for 24 h and the effects of various doses of the AO extract (5, 10, and 20 μg/mL) were monitored. (**A**) The inhibition of VSMC proliferation was determined in the presence of the AO extract was measured using crystal violet staining (n = 3). Images are presented at their original magnification (100×, scale bar: 100 μm). (**B**) Cytotoxicity of the AO extract on VSMCs was examined at three different concentrations (5, 10, and 20 μg/mL) and for various times using Annexin V-FITC/PI staining (a) and a colorimetric WST-1 assay (b) (n = 3). (**C**) The PDGF-induced phosphorylation of PLCγ1, STAT3, ERK1/2, Akt, p38, and JNK was measured using sodium dodecyl sulfate polyacrylamide gel electrophoresis (SDS-PAGE) and immunoblotting (a), using the total forms of each for normalization (n = 3) (b). The results were analyzed by densitometry and the values represent the mean ± SEM ratio relative to the PDGF-BB-stimulated controls. Significant differences from the PDGF-stimulated controls are indicated by asterisks: *P < 0.05, **P < 0.01, ***P < 0.001. The full size blot is shown in Supplementary Figure S3.

First, to assess the *in vitro* anti-proliferative activity of AO extract, we examined crystal violet staining assay. As shown in Fig. 1A, VSMC proliferation was suppressed in the presence of the AO extract at concentrations of 5, 10, and 20 μg/mL than in the stimulated control. To determine whether the anti-proliferative activity of AO extract was due to cytotoxicity, we examined the cytotoxicity at following treatment with 10, 30, and 50 μg/mL extract for various times using Annexin V-FITC/PI staining and a colorimetric WST-1 assay. The positive control (10% Triton-X) was found to be cytotoxic. By contrast, the AO extract did not appear to be cytotoxic, when measured by either apoptosis or necrosis (Fig. 1Ba and b).

In addition, in the phosphorylation of early signaling, including phospholipase C-γ1 (PLCγ), signal transducer and activator of transcription (STAT3), extracellular signal regulated kinase 1/2 (ERK 1/2), phosphatidylinositol 3-kinase-linked protein kinase B (AKT), p38, and c-Jun N-terminal kinase (JNK), between AO-treated cells and the PDGF-stimulated control was no significant difference (Fig. 1C). These results indicate that the anti-proliferative activity of AO results from downstream effects, such as on cell cycle progression, cell cycle-related proteins, and CKIs, rather than suppression of the early signaling transduction pathway.

### Suppression of cell cycle progression and the expression of cell cycle-related proteins by the AO extract

**Figure 2:**
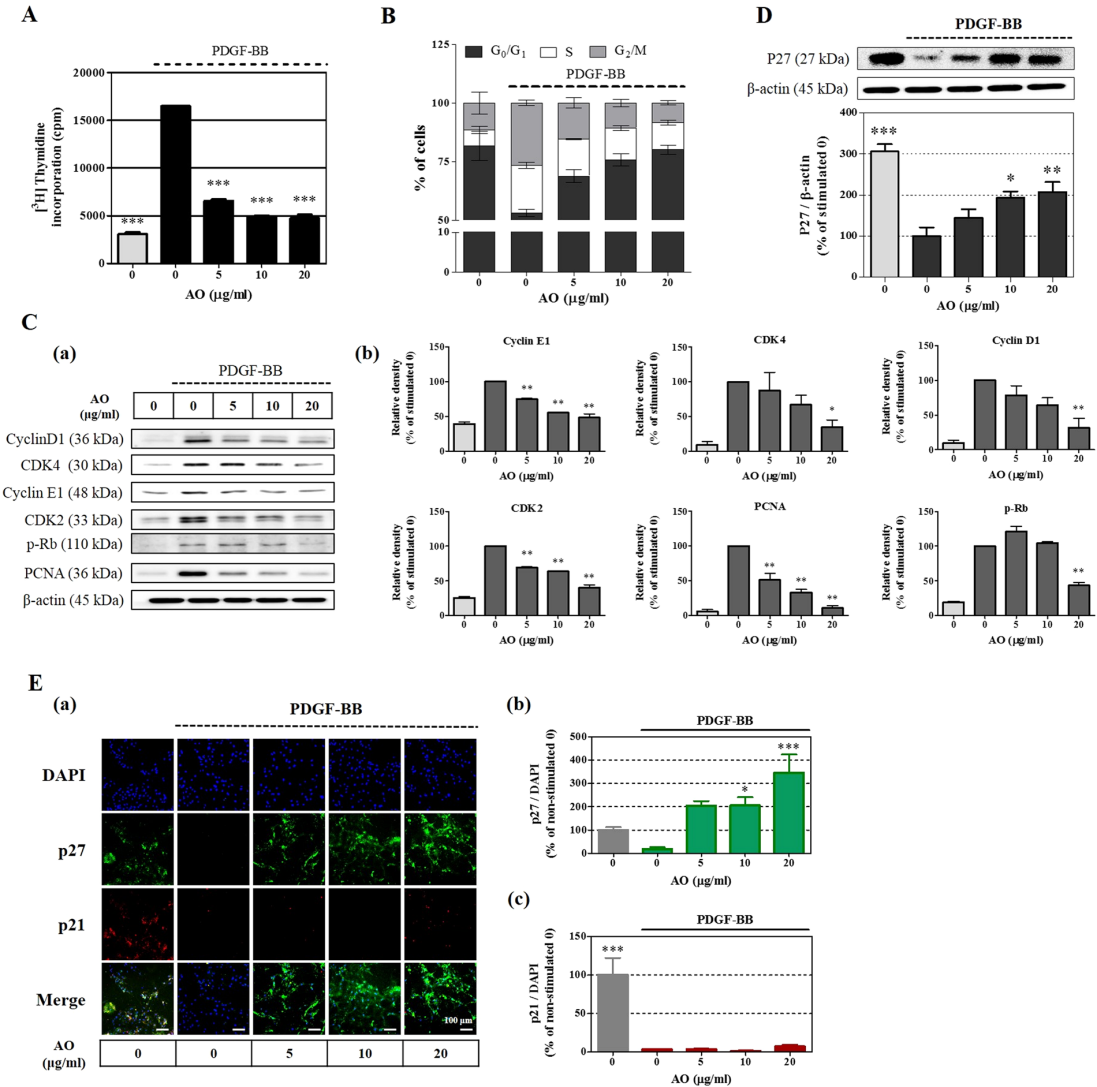
Effects of the AO extract on DNA synthesis and cell cycle regulatory proteins. Quiescent VSMCs cultured in serum-starved medium were stimulated with 25 ng/mL PDGF-BB, and the effects of the AO extract (5–20 μg/mL) on DNA synthesis (n = 3) (**A**) and the cell cycle progression (**B**) (n = 3) were assessed. (**C**) The effects of the AO extract on cell cycle regulatory proteins that were stimulated by PDGF-BB, including cyclin D1/E1, CDK2/4, Rb, and PCNA as negative regulatory molecules, was measured using SDS-PAGE followed by immunoblotting (n = 3) (a), using β-actin for normalization (b). (**D**) The inhibitory effects of the AO extract on p21^WAF1/CIP1^ and p27^KIP1^ expression were examined using SDS-PAGE followed by immunoblotting (a), using β-actin for normalization (b,c), and the results were analyzed using densitometry. (**E**) p21^WAF1/CIP1^ and p27^KIP1^ expression were also examined using an immunofluorescence assay (a), using the fluorescence levels of 4′,6-diamidino-2-phenylindole (DAPI) for normalization (b), and the levels of immunofluorescence were analyzed using densitometry; the values represent percentages relative to the stimulated control. Images are presented at their original magnification (200×, scale bar: 100 μm). The results are an average of three similar, independent experiments and are expressed as the mean ± SEM. Significant differences relative to the PDGF-stimulated controls are indicated by asterisks: *P < 0.05, **P < 0.01, ***P < 0.001. The full size blot is shown in Supplementary Figure S4.

Based on the above findings, we next investigated the effect of the AO extract on DNA synthesis and the expression of cell cycle-related proteins. The AO extract at concentration of 5, 10, and 20 μg/mL potently suppressed PDGF-induced DNA synthesis by at least 62% compared with the PDGF-stimulated control (Fig. 2A). Consistent with observed inhibition of DNA synthesis, the AO extract induced cell cycle arrest at the transition from the G_0_/G_1_ to the S phase of the cell cycle (Fig. 2B). These results indicate that the anti-proliferative effect of the AO extract is correlated with the expression of proteins that cause VSMC proliferation via transitions in the cell cycle, such as cyclin, CDK, retinoblastoma (Rb) protein, proliferation cell nuclear antigen (PCNA), and CKIs. Therefore, we next investigated the effect of AO extract on the activation of cell cycle-related proteins. As shown in Fig. 2C, overall, the AO extract significantly decreased the expression of cyclin D1, CDK4, cyclin E1, and CDK2 by suppressing Rb phosphorylation and PCNA expression. Moreover, among the CKIs, the expression of p27 increased by approximately two-fold compared with the PDGF-stimulated control in the presence of 10 μg/mL or more of AO extract (Fig. 2D), whereas the expression of p21 was not affected (Fig. S4). In addition, this result was confirmed using immunofluorescence staining in the VSMCs against p27 protein, which demonstrated that the AO extract at concentrations of 5, 10, and 20 μg/mL potently increased p27 expression by 204.6 ± 20.12, 206.0 ± 35.10, and 345.5 ± 78.64% compared with PDGF-BB-stimulated controls, respectively (Fig. 2E). These results indicate that the transition from the G_0_/G_1_ phase is suppressed through increased p27 expression in the presence of the AO extract.

### Determination of the active components in the AO extract

**Figure 3:**
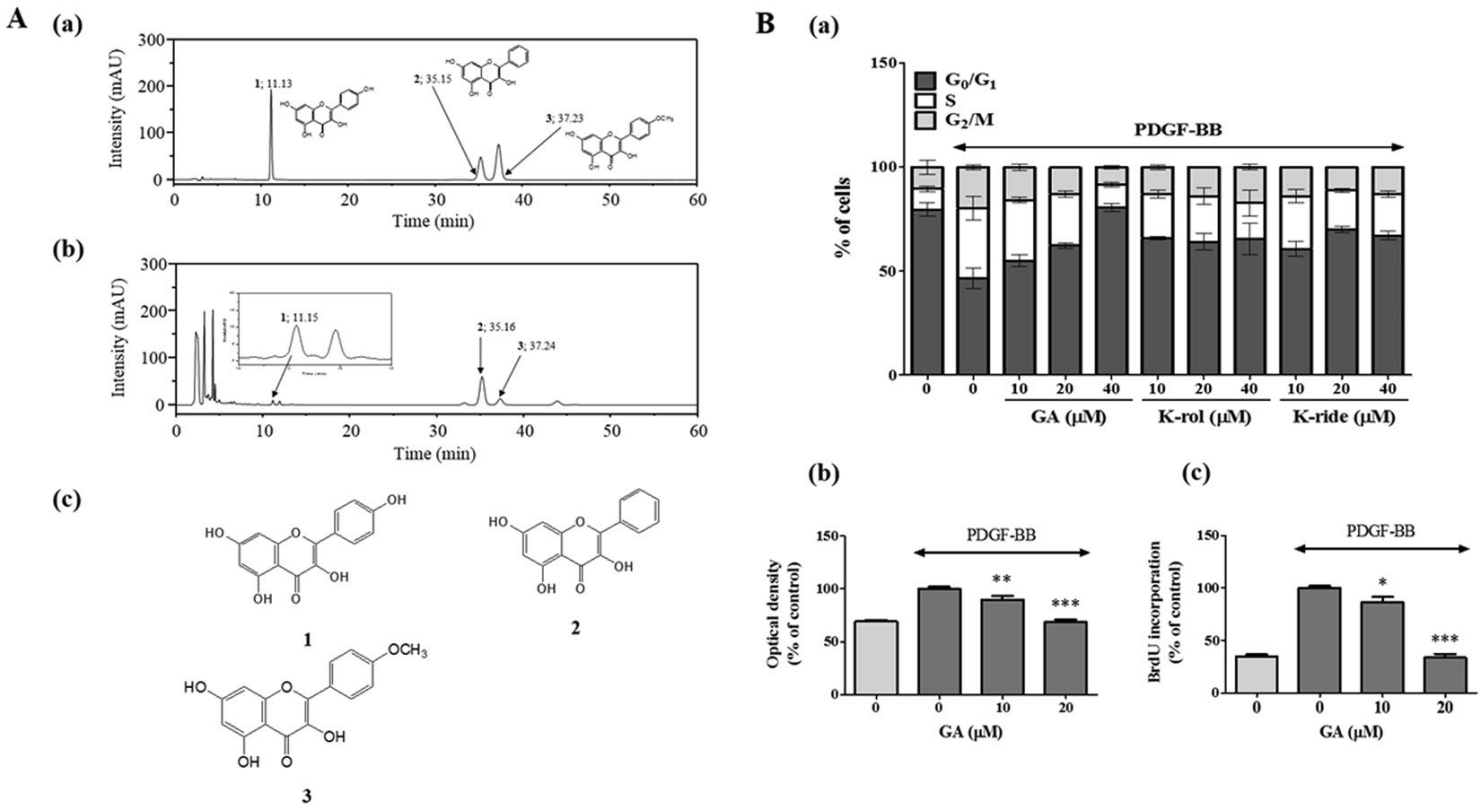
High-performance liquid chromatography (HPLC) analysis of the major compounds in the AO extract and determination of the active components. (**A**) HPLC chromatogram at 330 nm of a standard mixture including kaempferol (1, K-rol), galangin (2, GA), and kaempferide (3, K-ride) (a), and the AO extract prepared from a dry powder (b); the chemical structures are shown in (c). (**B**) The effects of GA, K-rol, and K-ride on cell cycle progression (n = 3) (a), and the effects of GA on VSMC proliferation (b) and DNA synthesis (c) (n = 4). The results were analyzed using densitometry, with the values representing the percentage relative to the PDGF-stimulated controls. The results are an average of three or more similar, independent experiments and are expressed as the mean ± SEM. Significant differences relative to the controls are indicated by asterisks: *P < 0.05, **P < 0.01, ***P < 0.001.

To determine the active components in the AO extract, we adjusted the UV wavelength of the chromatograms to 330 nm, according to the maximum UV absorption of the major standard compounds: kaempferol (K-rol), 336 nm; galangin (GA), 359 nm; and kaempferide (K-ride), 366 nm^20, 21^. The constituents of the AO extract were determined by HPLC with diode array detection (HPLC-DAD) analysis and each peak in the UV region was compared with that of the representative standard compounds. Single peaks in the AO extract were identified at similar retention times to the standard compounds: K-rol, 11.13 min cf. 11.15 min; GA, 35.15 min cf. 35.16 min; and K-ride, 37.23 min cf. 37.24 min (Fig. 3Aa and b). The chemical structures of each of these components are shown in Fig. 3Ac.

Therefore, we examined the effect of each of these components on PDGF-stimulated cell cycle progression. We found that only GA arrested the transition from the G_0_/G_1_ to the S phase of the cell cycle in a dose-dependent manner, matching the inhibitory pattern of the AO extract (Fig. 3Ba). Thus, to identify anti-proliferative effect of GA as an active component, we performed CCK assay and bromodeoxyuridine (BrdU) incorporation. GA significantly inhibited VSMC proliferation (Fig. 3Bb and c), this results have confirmed the potential of GA as the primary active component in anti-proliferative activity of the AO extract on VSMC proliferation.

### Verification of p27 as the target of action

**Figure 4:**
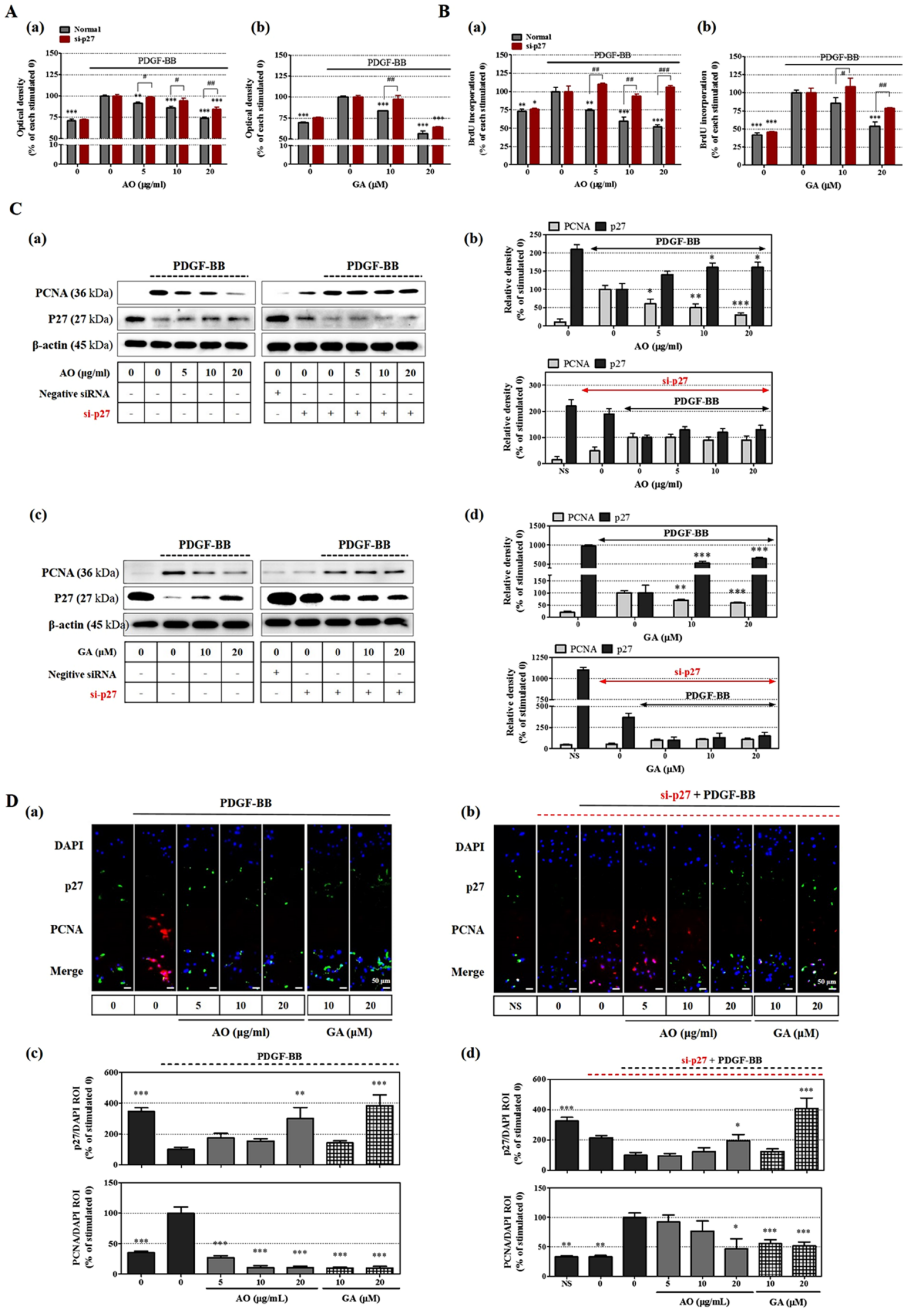
Identification of p27 up-regulation as the target of action in the anti-proliferative activity of the AO extract and GA. VSMCs were transfected with the siRNA of p27 (si-p27) for 6 h using TransIT-X2 and were then stimulated by 25 ng/mL PDGF-BB for 24 h. (**A**) The anti-proliferative effects and (**B**) the effects on BrdU incorporation of the AO extract (a,c) and GA (b,d) were determined on VSMCs in which p27 expression had been silenced at 450 nm using the WST-1 assay (n = 4) and on DNA synthesis (n = 4). (**C**) The inhibitory effects of the AO extract (a,b) and GA (c,d) on PCNA and p27^KIP1^ expression in the presence or absence of si-p27 were examined using SDS-PAGE followed by immunoblotting; β-actin was used for normalization and the results were analyzed using densitometry. (**D**) PCNA and p27^KIP1^ expression were also examined using an immunofluorescence assay in the presence (a) or absence (b) of si-p27, using the fluorescence levels of DAPI for normalization (c,d); the levels of immunofluorescence were analyzed using densitometry and the values represent percentages relative to the stimulated control (n = 5). Images are presented at their original magnification (400 × , scale bar: 50 μm). The results are an average of four or more similar, independent experiments and are expressed as the mean ± SEM. Significant differences relative to the PDGF-stimulated controls are indicated by asterisks: *P < 0.05, **P < 0.01, ***P < 0.001. The full size blot is shown in Supplementary Figure S5.

Our previous findings indicated that the AO extract inhibits VSMC proliferation by up-regulating p27 expression, with GA being the major active component. Therefore, to verify p27 protein as the target as action, we investigated the effect of the AO extract and GA by silencing the expression of p27. The AO extract inhibited VSMC proliferation and DNA synthesis in a dose-dependent manner; however, this inhibitory effect was significantly reduced at all treatment concentrations by silencing the expression of p27 (*p* < 0.05; Fig. 4Aa and Ba). Similarly, p27 silencing significantly reduced the inhibitory activity of GA on VSMC proliferation at a dose of 10 μM and on DNA synthesis at a dose of 10 and 20 μM (*p* < 0.05; Fig. 4Ab and Bb).

In addition, to confirm that these compounds have a direct effect on cell cycle-related protein, we conducted immunoblotting and an immunofluorescence assay while silencing the expression of p27. Negative control siRNA oligonucleotides (Bioneer CO., Daejeon, Korea) was used as negative control against siRNA of p27 protein. As shown in Fig. 4C, the AO extract not only showed a noticeably reduced effect on PCNA and p27 expression but also GA did not affect PCNA and p27 expression when p27 expression was suppressed by pretreatment with si-p27. Similarly, the immunofluorescence assay showed that both the AO extract and GA not only potently suppressed PCNA expression, but also significantly increased the fluorescence level of p27 to 229.80 ± 72.01 and 384.30 ± 68.52%, respectively, compared with the PDGF-induced control at concentrations of 20 μg/mL and 20 μM (Fig. 4Da and c). Furthermore, although the silencing of p27 expression reduced the inhibitory effect of the AO extract and GA against PCNA expression on VSMC proliferation, both the AO extract and GA increased the fluorescence level of p27 expression to 196.80 ± 40.89 and 408.40 ± 69.83%, respectively, at a high dose (Fig. 4Db and d).

Although, the complete silencing of p27 expression did not suppress the up-regulation caused by treatment with AO extract and GA, the silencing of p27 suppressed the increase in p27 expression caused by treatment with the AO extract and GA, and their inhibitory effects on PCNA expression also noticeably reduced from 72.80 ± 2.85, 89.16 ± 3.13, and 89.47 ± 2.48% to 7.83 ± 11.57, 23.27 ± 17.32, and 53.37 ± 16.95% at concentrations of 5, 10, and 20 μg/mL for the AO extract, and from 90.22 ± 1.58 and 90.05 ± 2.82% to 44.58 ± 6.41 and 48.54 ± 6.88% at concentrations of 10 and 20 μM for the GA (Fig. 4Dc and d). Since PCNA is a major gene that is synthesized through hyper-phosphorylation of the Rb protein during VSMC proliferation^22^, this suggests that expression of p27 protein is a major target of action in the anti-proliferative pathway of the AO extract and GA.

### Comparison with paclitaxel and rapamycin in neointima lesion formation *in vivo*

To identify the direct effects of these compounds in an animal model, we produced a neointima hyperplasia using cuff-injured animal model, which was treated with GA, paclitaxel (P) or rapamycin (R) dissolved in pluronic gel of cuff, or with the AO extract which was orally administered for 14 days, which was equivalent to the cuff-injured period.

**Figure 5:**
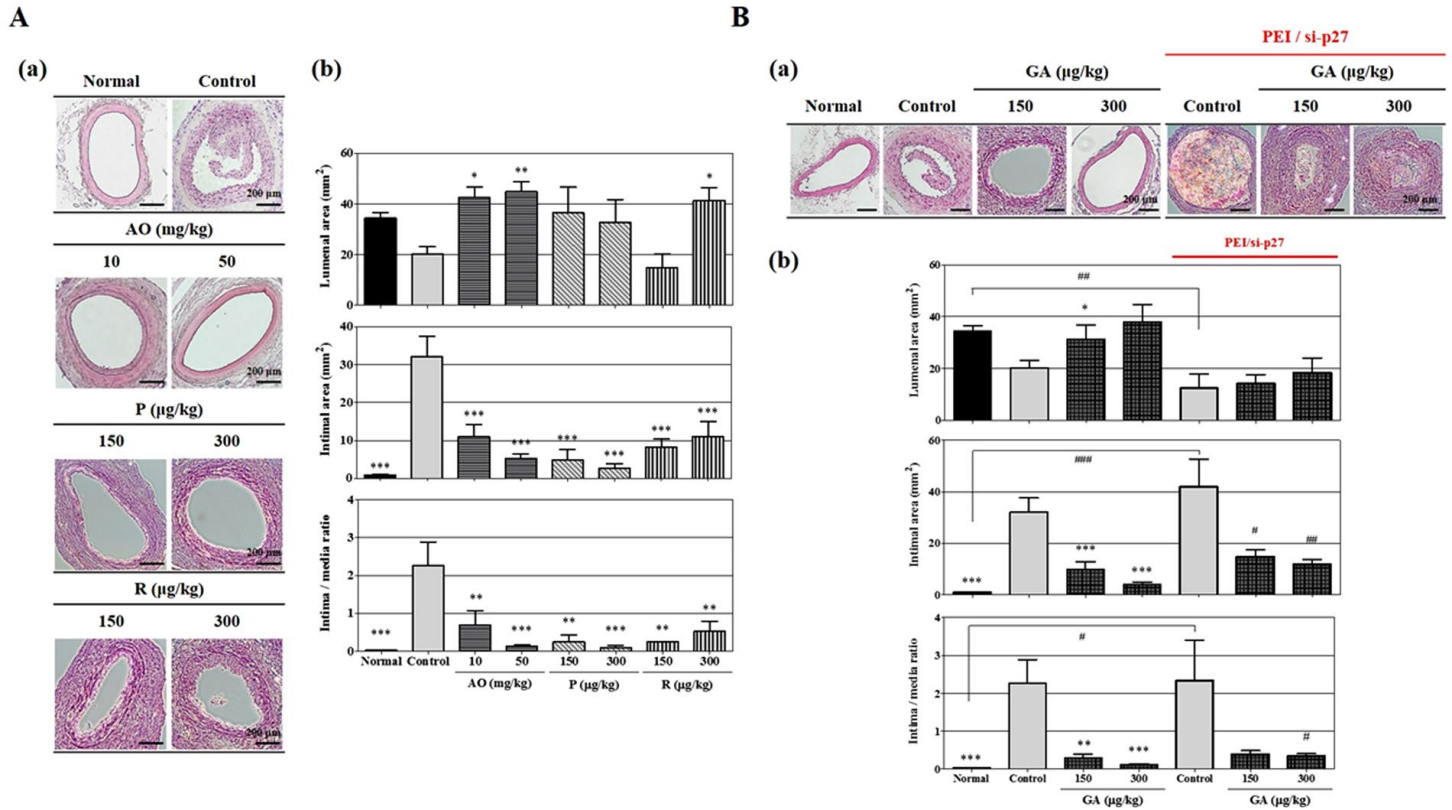
*In vivo* inhibitory effect of the AO extract and GA on VSMC proliferation in a cuff-injured neointimal hyperplasia model of the femoral artery. Rats were anesthetized with 2.5% isoflurane with supplementary O_2_ gas and the femoral artery was exposed by dissecting the surrounding tissues. Neointimal hyperplasia of the femoral artery was induced by applying a cuff filled with 30% pluronic F-127 gel for 14 days. (**A**) The effects of the AO extract (10 and 50 mg/kg) were examined using hematoxylin-eosin (H&E) staining on the cuff-injured neointimal hyperplasia model, using the existing clinical medicines paclitaxel and rapamycin (sirolimus) (150 and 300 μg/kg) as positive controls (a); the suppressive effects of the AO extract, paclitaxel, and rapamycin on the luminal area, intimal area, and intima/media ratio were then examined (n = 5) (b). (**B**) The effect of silencing p27 expression using *in vivo*-jetPEI® on the anti-proliferative actions of GA (150 and 300 μg/kg) in the cuff-injured neointimal hyperplasia model, showing H&E staining of the cuff-injured femoral artery (a), and its luminal area, intimal area, and intima/media ratio (b) (n = 6). Images are presented at their original magnification (300×; Aa and Ba), scale bar is 200 μm. The values are expressed as the mean ± SEM. Significant differences relative to the cuff-injured control and cuff-injured control pretreated with the PEI and si-p27 complex are indicated by asterisks: ^*,#^ *P* < 0.05, ^**,##^ *P* < 0.01, ^***,###^ *P* < 0.001.

The AO extract significantly increased the luminal area to 209 ± 4.12 and 220.72 ± 4.10% at doses of 10 and 50 mg/kg compared with the cuff-injured control, and rapamycin increased it to 204.38 ± 4.92% at a dose of 300 μg/kg (Fig. 5Aa and b), whereas paclitaxel did not significantly affect the luminal area. By contrast, the AO extract, rapamycin, and paclitaxel all potently reduced the intimal area and the intima/media ratio. These findings not only confirm the effects of these standard clinical medicines for treating restenosis and atherosclerosis, but also show that the AO extract has a large effect, even if it was administered orally at a higher dose.

Next, we also investigated the effects of GA to confirm the target of action *in vivo*. We found that cuff-injured animals in which p27 expression had been silenced developed a smaller luminal area than non-PEI/si-p27-cuff-injured animals by increasing neointima formation from 31.29 ± 5.65 and 38.12 ± 6.64 mm^2^ to 14.13 ± 3.58 and 18.48 ± 5.67 mm^2^ at doses of 150 and 300 μg/kg (Fig. 5Ba and b). However, GA treatment led to only a very weak reduction of inhibitory activity on intimal area and intima/media ratio of animals in which p27 had been silenced, however, despite PEI/si-p27-cuff-injured animals exhibiting more potent intima formation than non-PEI/si-p27-cuff-injured animals in control group (Fig. 5Bb).

These results indicate that, together with the clinical medicines paclitaxel and rapamycin, the AO extract and GA evidently inhibit the induction of restenosis. In addition, these results suggest that p27 expression affects as a major target of anti-proliferative effect of GA.

### Determination and verification of the target of action *in vivo*

**Figure 6:**
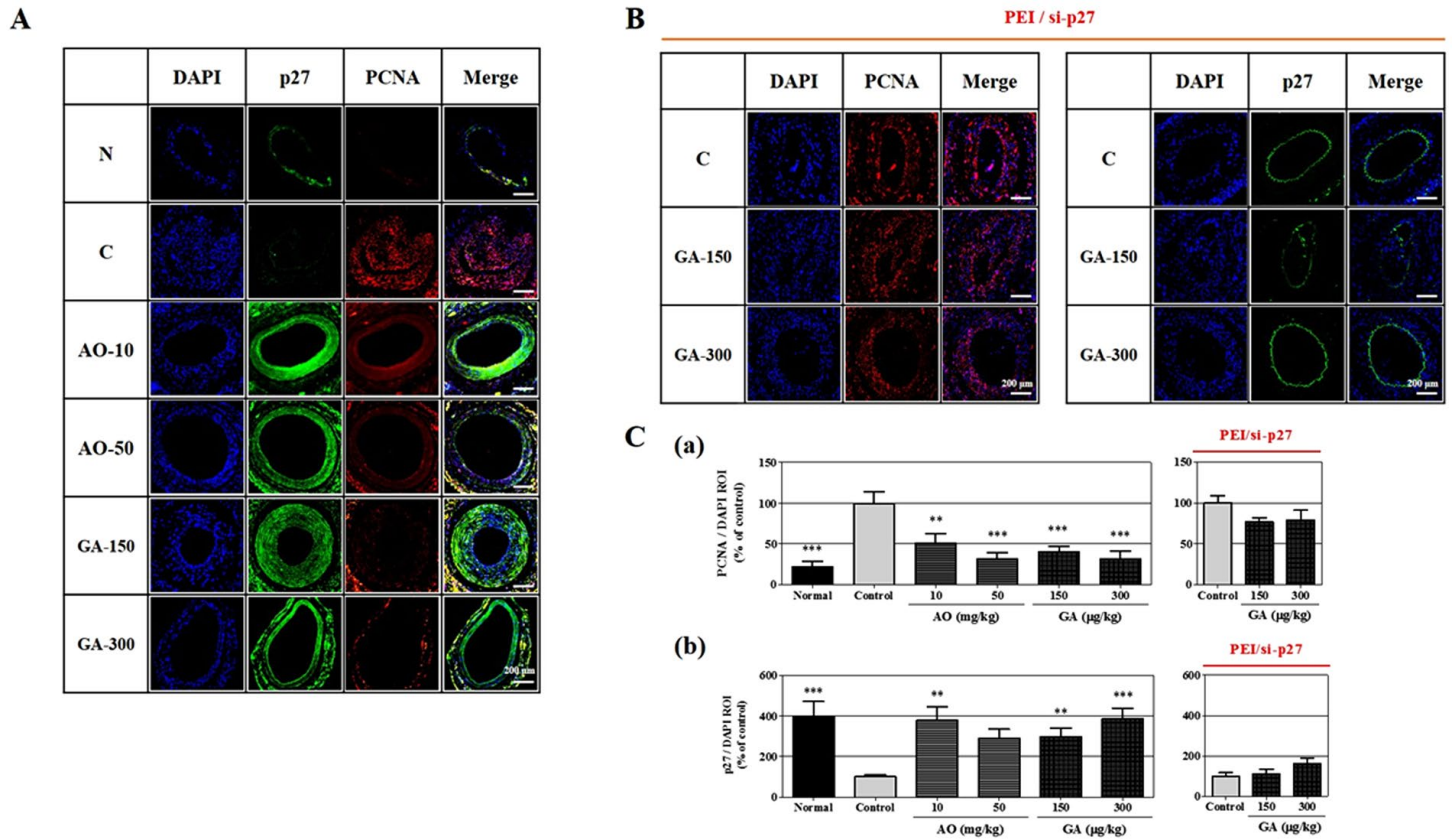
Effects of the AO extract and GA on PCNA and p27 expression in a cuff-injured neointimal hyperplasia model of the femoral artery. The immunofluorescence levels of PCNA and p27 expression were examined in the femoral artery. (**A**) The suppression of PCNA expression and the up-regulation of p27 expression by the AO extract (10 and 50 mg/kg) and GA (150 and 300 μg/kg) were detected using immunofluorescence staining. (**B**) The effect of silencing p27 expression on the PCNA suppression and p27 up-regulation by GA (150 and 300 μg/kg). (**C**) Comparison of the fluorescence levels expressed PCNA (a) and p27 (b) as normalized values of DAPI (n = 5). Images are presented at their original magnification (300×; **A** and **B**), scale bar is 200 μm. The values are expressed as the mean ± SEM. Significant differences relative to the cuff-injured control are indicated by asterisks: **P < 0.01, ***P < 0.001.

To verify whether p27 protein is a major target of action in the anti-proliferative activity of GA *in vivo*, we examined the effect of PCNA expression with respect to increased or silenced p27 expression in a cuff-induced neointima animal model. PCNA expression was inhibited to 48.56 ± 10.95 and 68.50 ± 7.65% at doses of 10 and 50 mg/kg of orally administered AO extract, respectively, and to 60.11 ± 7.15 and 68.02 ± 9.22% at doses of 150 and 300 μg/kg of GA (Fig. 6A and C). Moreover, the immunofluorescence level of p27 expression increased to 378.40 ± 66.65 and 289.30 ± 44.39% at doses of 10 and 50 mg/kg of AO extract, respectively, and to 297.50 ± 41.27 and 386.00 ± 52.68% at doses of 150 and 300 μg/kg of GA, respectively (Fig. 6A and C). These results indicate that the up-regulation of p27 expression via treatment with GA causes an anti-proliferative effect via the suppression of PCNA expression.

Additionally, to identify the impact of PCNA expression, we silenced p27 expression by pretreating cells with PEI/si-p27. As shown in Fig. 6B and C, PCNA expression was reduced to 23.21 ± 5.01 and 20.74 ± 11.97% at doses of 150 and 300 μg/kg of GA, respectively, compared with the cuff-injured control group, while the up-regulation of p27 expression decreased to 111.00 ± 21.91 and 162.90 ± 24.75% compared with the GA treatment on non-PEI/si-p27 animals. These results show that p27 expression is a major target of the anti-proliferative effect of GA on neointima formation, and that this dependently interacts with PCNA expression.

## Discussion

This study clearly demonstrates that GA has potential as an alternative to existing clinical medicines for the treatment of restenosis and atherosclerosis. Both GA and the AO extract from which it was isolated inhibited VSMC proliferation by arresting the transition from the G_0_/G_1_ phase to the S phase of the cell cycle by up-regulating p27^KIP1^ (Figs 1–4), and these effects were confirmed in an animal model *in vivo* (Figs 5 and 6).

It has been well established that abnormal VSMC proliferation in the arteries plays a key role in the development of neointima, having particular pathophysiological significance in the formation of restenosis and atherosclerotic plaques^23^. The exposure of VSMCs to growth factors and cytokines following an inflammatory response of the vascular endothelial cells causes their proliferation and activates early signals, such as PLCγ1, STAT3, mitogen-activated protein kinase (MAPK), and AKT^24, 25^. Therefore, we first examined the effect of the AO extract on VSMCs treated with PDGF-BB, which is a major inducer of VSMC proliferation and migration^26^. We found that the AO extract potently suppressed PDGF-BB-induced VSMC proliferation but had no suppressive effect on early signal phosphorylation, indicating that this inhibitory effect was related to cell cycle-related signal transduction (Fig. 1).

Cell cycle progression in VSMCs is positively regulated by CDK/cyclin complexes and negatively regulated by CKIs^27, 28^. The AO extract potently inhibited DNA synthesis in a dose-dependent manner, arresting the transition from the G_0_/G_1_ to the S phase and significantly suppressing the proteins that are related to this transition. Rb hyper-phosphorylation is induced by the expression of cyclin/CDK complexes, leading to the release of E2F and allowing the VSMCs to progress to the S phase^29^ (Fig. 2A–C). The AO extract significantly inhibited Rb phosphorylation and perfectly suppressed PCNA expression (Fig. 2C). In addition, the AO extract up-regulated the expression of p27^KIP1^ by approximately two-fold compared with the PDGF-stimulated control (Fig. 2D), but had no significant effect on other proteins such as p21^WAF1/CIP1^ (Fig. S4). p27 is expressed in healthy or normal arteries, but is down-regulated in cases of neointima hyperplasia, which is accompanied by an increased intimal VSMC proliferation and intima/media ratio^30, 31^. The combined use of an immunofluorescence assay, and p27 silencing showed that the AO extract significantly increased the fluorescence level of p27 expression to a maximum of 345.50%, while p21 expression was suppressed at all doses compared with the normal non-stimulated control (Fig. 2E).

HPLC-DAD analysis showed that the AO extract contained the flavonoids K-rol, GA, and K-ride (Fig. 3A). Since *Alpinia officinarum* Hance belongs to the ginger family, we also expected to detect quercetin in the AO extract, as this is present in many vegetable, fruits, grains, and leaves^32, 33^; however, we did not detect this flavonoid (Tables S1 and S2). Among the detected components, GA had the highest content (1.69 mg/g of AO extract) (Table S2 and Figure S2) and was the only compound that arrested the cell cycle transition of the G_0_/G_1_ phase in a dose-dependent manner (Fig. 3Ba). Furthermore, GA perfectly inhibited VSMC proliferation and DNA synthesis at a dose of 20 μM (Fig. 3Bb and c), indicating that it may be the active component in the anti-proliferative activity of the AO extract.

The degradation of p27^KIP1^ is important in the cell cycle progression from the G_0_/G_1_ to S phase and is also a requirement for the resulting phosphorylation of Rb protein via the activation of cyclin/CDK complexes^30, 34^, and so the up-regulation of p27 expression was considered an efficient target for treatment of restenosis and atherosclerosis. The anti-proliferative activity of both the AO extract and GA significantly decreased when p27 expression was silenced, indicating that overexpression of p27 is a major target of both the AO extract and GA (Fig. 4A). We also found that while both the AO extract and GA significantly inhibited PCNA expression under normal circumstances, this effect was markedly reduced when p27 expression was silenced, and confirmed these findings using an immunofluorescence assay (Fig. 4C and D). Thus, it is clear that p27 expression is a major target of these compounds and that GA is the major active component of the AO extract.

It is widely accepted that the overexpression of p27^KIP1^ is an important target for treatment in an animal model of neointima formation. It has previously been shown that a reduction in intimal formation and the intima/media (I/M) ratio in pig arteries induces vascular injury^31^, and that the delivery of adenovirus vectors encoding p27^KIP1^ suppresses neotintima hyperplasia in rat carotid arteries exhibiting angioplasty^35^. In the present study, oral administration of the AO extract resulted in a two-fold or greater increase in the luminal area of cuff-injured femoral arteries *in vitro*, as did rapamycin, whereas paclitaxel had no significant effect (Fig. 5A). In addition, the intimal area was significantly reduced following treatment with the AO extract, paclitaxel, and rapamycin at all doses, which relates to the degree of neointima formation, and the I/M ratio was also clearly reduced (Fig. 5A). GA also exhibited an anti-proliferative effect on the VSMCs *in vivo*, including an increase in the luminal area, suppression of intimal growth, and a lowering of the I/M ratio (Fig. 5B). Interestingly, the silencing of p27 expression caused a reduction in the intimal area in the cuff-injured control group by consolidating the abnormal proliferation of the VSMCs, while the GA-treated group also experienced a reduced increase in the luminal area (Fig. 5B). However, GA significantly suppressed intimal formation and the I/M ratio even in the presence of p27 silencing, suggesting that its anti-proliferative activity was not suppressed by p27 silencing.

Hence, we needed to ascertain whether p27 expression was perfectly silenced in this animal model. Treatment with the AO extract and GA dose-dependently increased p27 expression compared with the control group, and also potently suppressed PCNA expression (Fig. 6A). However, surprisingly, this inhibitory effect by GA on PCNA expression disappeared when p27 overexpression was silenced (Fig. 6B). The importance of p27 expression in regulating neointima hyperplasia, including atherosclerosis and restenosis, has been well-established in previous studies, which have demonstrated the effects of DES containing rapamycin in the suppression of restenosis following angioplasty^36^ and the attenuation of neointimal hyperplasia by the transfer of the p27 gene^35^. Thus, the findings of the present study demonstrate that the up-regulation of p27 expression is an important component of the preventative effects of GA on neointima formation caused by the abnormal proliferation of VSMCs.

In conclusion, treatment with the AO extract and GA led to the inhibition of VSMC proliferation by arresting the cell cycle transition from the G_0_/G_1_ phase, resulting in up-regulation of p27 expression. Moreover, the substantial suppressive effect of these compounds in neointimal hyperplasia was also demonstrated *in vivo*, where they had an inhibitory effect on abnormal proliferation of the VSMCs by causing the overexpression of p27, which was a major target of action. These findings indicate that GA may have potential as an alternative agent for DES with fewer side effects than existing clinical medicines.

Although the effect of the AO extract and GA on neointima formation caused by the abnormal proliferation of VSMCs was demonstrated in this study, the initial progression of atherosclerosis and neointima hyperplasia occurs via the inflammation response of endothelial cells (ECs), which constitute the main cell types together with VSMC within the vasculature^37, 38^. Moreover, a normal ECs is crucial due to its participation in the regulation of vascular tone and its role in suppressing intimal hyperplasia by inhibiting inflammation, thrombosis, and VSMC proliferation and migration^39, 40^. Hence, because the effect of the AO extract and GA in ECs should identify, we are performing as further study, separately.

## Methods

### Materials

*Alpinia officinarum* Hance (AO) was purchased from an herb market (Yeongcheon, Republic of Korea) and then was identified by Dr. Ki Hwan Bae of the College of Pharmacy, Chungnam National University (Daejeon, Republic of Korea); a voucher specimen has been stored in the herbal bank at the Korea Institute of Oriental Medicine (Daejeon, Korea). Galangin (GA), paclitaxel, sirolimus (rapamycin) and kaempferol (K-rol) were purchased from Sigma-Aldrich (St. Louis, MO, USA). Kaempferide (K-ride) was purchased from ChemFaces (Wuhan, Hubei, China). The purity of all the chemical reference substances was greater than 95%. High-performance liquid chromatographic (HPLC) grade acetonitrile and methanol were procured from J.T. Baker Inc. (Philipsburg, NJ, USA), and formic acid was purchased from Wako (≥99.5%, Wako Pure Chemical Industries, Ltd., Osaka, Japan). Ultrapure water (UW) was prepared using the Puris-Evo UP Water system with Evo-UP Dio VFT and Evo-ROP Dico20 (Mirae ST Co., Ltd., Anyang, Gyeonggi-do, Korea). UW was prepared to have a resistivity of 18.2 MΩ cm^−1^ (Puris, Esse-UP Water system, Mirae St Co., Anyang, Korea). Fetal bovine serum (FBS) and phosphate-buffered saline (PBS) were purchased from HyClone (Logan, UT, USA). Distilled water was filtered through a 0.45-µm membrane filter from ADVANTEC (Tokyo, Japan) before analysis. Dulbecco’s modified Eagle’s medium (DMEM) was purchased from Lonza (Walkersville, MD, USA). Trypsin/EDTA and penicillin/streptomycin were purchased from Gibco (Grand Island, NY, USA). Anti-phospho-ERK1/2, anti-ERK1/2, anti-phospho-PLCγ1, anti-phospho-STAT3, anti-STAT3, anti-phospho-p38, anti-p38, anti-phospho-Akt, anti-Akt, anti-phospho-JNK, anti-JNK, anti-CDK2, anti-CDK4, anti-cyclin D1, anti-cyclin E1, anti-phospho- Rb, anti-PCNA, anti-p21, anti-p27, and anti-β-actin antibodies were purchased from Cell Signaling Technology Inc. (Beverly, MA, USA). The Cell Counting Kit-8 (CCK-8) was purchased from Dojindo Molecular Technologies (Rockville, MD, USA). *In vivo*-jetPEI was purchased from Polyplus transfection (Illkirch, France). Platelet-derived growth factor (PDGF)-BB was obtained from PEPROTECH Co. (Rocky Hill, NJ, USA). All other chemicals were of analytical grade.

### AO extract preparation

Dried *Alpinia officinarum* Hance (50.0 g) was placed in 1 L of distilled water and heated for 3 h at 115 °C using a Gyeongseo Extractor (Cosmos-600, Inchon, Korea). The extracted solution was filtered using a standard test sieve (150 μm; Retsch, Haan, Germany), and then freeze-dried and maintained in a desiccator at 4 °C until use.

### Cell culture

VSMCs, a primary cell type isolated from rat aorta via enzymatic dispersion^41^, were obtained from Biobud Co. (Seongnam-si, Gyeonggi-do, Korea). VSMCs were cultured in DMEM (supplemented with 10% FBS, 100 IU/mL penicillin, 100 μg/mL streptomycin, 8 mM HEPES, and 2 mM L-glutamine) at 37 °C in a humidified atmosphere of 95% air and 5% CO_2_. The purity of the cultures was confirmed based on immunocytochemical localization of α-smooth muscle actin. Our experiment used the VSMCs from passages 5 to 7.

### VSMC proliferation

VSMCs proliferation was investigated using the crystal violet assay, which measures the levels of crystal violet dye in cells^42^. In brief, VSMCs were seeded into 96-well culture plates at 4 × 10^4^ cells/mL and then cultured in complete media (DMEM containing 10% FBS) at 37 °C for 24 h. After reaching approximately 70% confluence, VSMCs were incubated with serum-free medium for 24 h, treated with AO extract or GA at various concentrations for another 24 h (in newly made serum-free medium), and stimulated with PDGF-BB (25 ng/mL). Then, after 24 h, the VSMCs was washed with ice-cold PBS and then fixed in 4% paraformaldehyde (in PBS). The cells were then stained with 0.5% crystal violet for 15 min, washed with deionized water, and observed by microscopy (Nikon Eclipse Ti; Nikon instruments Inc., Melville, NY, USA).

### Cell cytotoxicity

Cytotoxicity was assessed by treating VSMCs with AO extract, and then using a colorimetric WST-1 assay to count cells. The cells were exposed to AO extract at various concentration, or 10% Triton X-100 as a cytotoxic control, for various time periods. WST-1 reagent was added to the medium, and the cells were incubated for 1 h. Absorbance density was measured at 450 nm using a microplate reader. In addition, we also tested the cytotoxicity of the AO extract using an Annexin V-FITC/PI staining kit. The cell culture conditions were the same as described for the VSMCs proliferation assay. Following stimulation with PDGF-BB (25 ng/mL) for 24 h, VSMCs were harvested by centrifugation and then fixed with 70% ethanol for 48 h. Finally, the centrifuged pellets were stained with Annexin V-FITC/PI solution at room temperature for 15 min, according to manufacturer’s instructions, and each cell nucleus was counted using flow cytometry (Gallios; Beckman Coulter Inc., Indianapolis, IN, USA).

### DNA synthesis

DNA synthesis was monitored using a [^3^H]-thymidine incorporation assay, as previously described^43^, using the same cell culture conditions as outlined above for the cell proliferation assay. [^3^H]-thymidine (2 μCi/mL) was bound for 4 h before harvesting under PDGF-BB-stimulated conditions (25 ng/mL) in serum-free medium. The reaction was stopped by aspirating the medium and subjecting the cultures to a series of washes with phosphate-buffered saline (PBS) containing ethanol/ether (1:1, v/v) and 10% trichloroacetic acid on ice. The acid-insoluble products containing [^3^H]-thymidine were extracted in 250 µl of 0.5 M NaOH/well, and this solution was then combined with 3 mL scintillation cocktail (Ultimagold; Packard Bioscience, Meriden, CT, USA) and measured using a liquid scintillation counter (LS3801; Beckman, Düsseldorf, Germany). To identify the active components of the AO extract, we used a bromodeoxyuridine (BrdU) colorimetric kit (Roche, Basel, Switzerland) to investigate the effect of GA on DNA synthesis during VSMC proliferation. After stimulation with PDGF-BB (25 ng/mL) for 24 h, VSMCs were labeled with BrdU, according to the manufacturer’s instructions, and BrdU-labeled cells were measured using a microplate reader.

### Cell cycle analysis

Cell cycle progression was examined as previously described^43^ using the same cell culture conditions as outlined above for the cell proliferation assay. Following stimulation with PDGF-BB (25 ng/mL) for 24 h, VSMCs were trypsinized and centrifuged at 749 × *g* for 7 min, and the centrifuged pellets were suspended in 1 mL PBS (1×) (repeated twice). The cells were then fixed with 70% ethanol for 48 h. The fixed cells were briefly stirred and centrifuged at 12400 × *g* for 5 min, following which the ethanol was removed and the pellets were stained with 500 μL propidium iodide (PI) solution (50 μg/mL PI in sample buffer containing 100 μg/mL RNase A). Each sample was incubated at room temperature for 1 h. The PI-DNA complex in each cell nucleus was measured using flow cytometry, with the nuclear DNA content of each cell being proportional to the PI fluorescence intensity. The number of cells in the G_0_/G_1_, S, and G_2_/M phases was then determined using flow cytometry and the Kaluza analysis software (Beckman Coulter Inc., Indianapolis, IN, USA).

### Immunoblotting

Immunoblotting was performed to measure the expression of proteins, as previously described^43^. VSMCs were stimulated with PDGF-BB (25 ng/mL) for 5 min for ERK1/2 and PLCγ1, 10 min for JNK and p38, and 15 min for STAT 3 and Akt phosphorylation assays. To measure the expression of CDK2, CDK4, cyclin D1, cyclin E_1_, p21, p27, and proliferating cell nuclear antigen PCNA, and the phosphorylation of Rb, VSMCs were stimulated with PDGF-BB (25 ng/mL) for 24 h. The levels of each protein were normalized to the levels of β-actin or the respective total protein. Band intensities were quantified using Scion Image for Windows (Scion Corp., Frederick, MD, USA).

### Immunofluorescence staining

VSMCs were cultured in 24-well plates on cover slips (8 × 10^4^ cells/mL), stimulated with PDGF-BB (25 ng/mL) for 24 h, fixed with PBS containing 2% paraformaldehyde for 10 min, and then blocked with 5% bovine serum albumin in PBS for 1 h at room temperature. The cells were incubated with primary antibodies (anti-PCNA, anti-p21 or anti-p27) for 2 h, and then stained with Alexa Fluor 488 and 555-conjugated secondary antibodies (Cell Signaling Technology Inc., Beverly, MA, USA). The cells were mounted in 4′,6-diamidino-2-phenylindole (DAPI) solution for 10 min and then observed by fluorescence microscopy.

### siRNA transfection

The siRNA for p27^KIP1^ (si-p27) and negative control siRNA oligonucleotides were synthesized by Bioneer Co. (Daejeon, Korea). The target sequences of si-p27 are 5′-AGU ACA CUU GAU CAC UGA A(dTdT)-3′ and antisense 5′-UUC AGU GAU CAA GUG UAC U(dTdT)-3′. si-p27 transfection was performed using TransIT-X2 of Mirus BIO LLC (Madison, WI, USA), according to the manufacturer’s instructions. VSMCs were treated with the siRNA (50 nM) for 6 h, following which the medium including the siRNA was removed and replaced with serum-free medium. Silencing target protein activation was identified via a cell counting kit, BrdU incorporation, immunoblotting, and immunofluorescence staining.

### Chromatographic systems

Each standard stock was prepared by dissolving GA, K-rol or K-ride in 100% methanol at 1 mg/mL. The solution was then filtered through a 0.2-mm syringe membrane filter (Whatman Ltd, Maidstone, UK) and the sample stock solution (50 mg in 100% methanol) was extracted using ultrasonic vibration at room temperature for 30 min. All standard and sample solutions were stored at 4 °C before analysis.

Separation was performed in a high-performance liquid chromatography (HPLC) system (Dionex Ultimate 3000; Thermo Fisher Scientific) comprising a pump, auto sampler, column oven, and diode array UV/VIS detector. Chromatograms were recorded using the Chromeleone software (version 7) system. The components of the AO extract were separated on a Luna C_18_ column (4.6 × 250 mm, 5 μm; Agilent, CA, USA) at 40 °C, with an injection volume of 10 μL and a detective wavelength set at 330 nm. The mobile phase, which consisted of 0.05% phosphoric acid and acetonitrile, was run at a flow rate of 1.0 mL/min. The isocratic elution program was set at 35% acetonitrile for 60 min^20^.

### Femoral artery cuff-injured model

The animal studies were carried out in accordance with the Korea Institute of Oriental Medicine Care Committee Guidelines and the dictates of the National Animal Welfare Law of Korea. This animal study was approved by the Korea Institute of Oriental Medicine Care and Use Committee (Protocol # 15-093).

Sprague Dawley (SD) rats (7 weeks old) were purchased from Sam-Tako Animal Co. (Osan, Korea) and housed under the following conditions: temperature, 22 ± 1 °C; humidity, 55 ± 5%; photoperiod: 12 h light:12 h dark). They were provided with a standard diet and water ad libitum.

Surgery of the neointimal hyperplasia animal models was performed using the cuffs of polyethylene tubes (PE-160, 4 mm length, 1.14 mm ID, 1.57 OD; Becton Dickinson, Franklin Lakes, NJ, USA), as previously described^44^. The rats were anesthetized with 2.5% isoflurane (Hana Pharm Co., Seoul, Korea) with supplementary O_2_ gas and the femoral artery was exposed by dissecting the tissues surrounding it. A cuff was loosely placed around the femoral artery, and the space between the cuff and femoral artery was then filled with 30% pluronic F-127 gel (Sigma-Aldrich, St. Louis, MO, USA) containing the appropriate concentration of dissolved GA, paclitaxel or rapamycin. The AO extract was orally administered for 14 days, which was equivalent to the cuff-injured period. The control group did not have a cuff fitted while the cuff-injured control group was filled with 30% pluronic F-127 gel with no drug.

### Pretreatment with the PEI-siRNA complex

p27 siRNA (si-p27) and *in vivo*-jetPEI® (Polyplus transfection; Illkirch, France) were combined into a complex, according to the manufacturer’s instructions, as previously described^45, 46^. si-p27 solution in 5% glucose (150 μg/mL) was mixed with *in vivo*-jetPEI and then allowed to stand for at least 30 min at room temperature. This complex was injected intravenously four times over 14 days.

### Morphometric analysis

The femoral artery was harvested from each rat 14 days after the cuff injury, as previously described^44^. Briefly, 14 days after placing the cuff, the rats were anesthetized and subsequently perfused by cardiac puncture with red blood cell (RBC) lysis buffer (15 mM NH_4_Cl, 10 mM NaHCO_3_, and 0.1 M ethylenediaminetetraacetic acid [EDTA]) and saline, and then fixed with 10% formalin. All tissues except the femoral artery were then removed and the femoral artery was embedded in paraffin, sectioned (5 μm), and stained with hematoxylin-eosin (H&E). The intima, media, and lumina areas were then quantified using Scion Image.

### Statistical analysis

The data are expressed as the mean ± SEM values. A one-way analysis of variance was used for multiple comparisons (GraphPad, San Diego, CA, USA). Dunnett’s test was applied if a significant difference was observed among the treated groups. *P* < 0.05 was considered to be statistically significant.

## Electronic supplementary material

Supplementary Information
