# Supplementary material for: Application of galangin, an active component of Alpinia officinarum Hance (Zingiberaceae), for use in drug-eluting stents
Source: Sci Rep. 2017 Aug 15;7:8207. doi: 10.1038/s41598-017-08410-2 (PMC5557749; doi:10.1038/s41598-017-08410-2)
Supplement: Supplementary file 1 — Supplementary Information [file 41598_2017_8410_MOESM1_ESM.pdf]

Supplementary Information

Application of galangin, an active component of *Alpinia officinarum* Hance (Zingiberaceae), for use in drug-eluting stents

Jung-Jin Lee[1], Ji-Hye Lee[1], Nam-Hui Yim[1], Joo-Hui Han[2], Jin Yeul Ma [1,*]

- Supplementary Table S1
- Supplementary Table S2
- Supplementary Figure S1
- Supplementary Figure S2
- Supplementary Figure S3
- Supplementary Figure S4
- Supplementary Figure S5

*Corresponding author: Jin Yeul Ma, PhD, Korea Medicine (KM) Application Center, Korea Institute of Oriental Medicine, Daegu, 701-300, Republic of Korea.

Phone: (+82) 53-940-3811; Fax: (+82) 53-940-3899; E-mail address: jyma@kiom.re.kr

Results

In the present study, the UV wavelength of chromatograms was adjusted based on the maximum UV absorption of major standard compounds: quercetin (1); 336 nm, kaemferol (2); 322 nm, galangin (3); 359 nm and kaempferide (4); 366 nm, therefore, they were analyzed at 330nm. The constituents of AO were determined by HPLC-DAD analysis and each peak of UV spectra was compared with that of representative standard compounds. As shown in figure 2 and table 1, HPLC-DAD analysis revealed that single peaks of each chemical standard contained in the extracts of AO were identified at similar retention times (t_R): kaemferol; 11.00 min, baicalein; 13.12 min, galangin; 34.80 min and kaempferide; 36.84 min, compared to those of standard compounds: kaemferol; 10.90 min, baicalein; 13.09 min, galangin; 34.80 min and kaempferide; 36.89 min.

We expected to analyze with quercetin, as a flavonoid which exists in many vegetable, fruits, grains and leaves, because AO aqueous extract is the ginger family,[1,2] but did not detected (Table 2). Among the detected components, the result of quantitative analysis showed galnagin as the most content of 1.69 mg/g (of AO extract).

Methods

Materials. *Alpinia officinarum* Hance (AO) was purchased from an herb market (Yeongcheon, Republic of Korea) and then was identified by Dr. Ki Hwan Bae of the College of Pharmacy, Chungnam National University (Daejeon, Republic of Korea); a voucher specimen has been stored in the herbal bank at the Korea Institute of Oriental Medicine (Daejeon, Korea). Galangin (GA), quercetin, paclitaxel, sirolimus (rapamycin) and kaempferol (K-rol) were purchased from Sigma-Aldrich (St. Louis, MO, USA). Kaempferide (K-ride) was purchased from ChemFaces (Wuhan, Hubei, China)[3,4]. The purity of all the chemical reference substances was greater than 95%. High-performance liquid chromatographic (HPLC) grade acetonitrile and methanol were procured from J.T. Baker Inc. (Philipsburg, NJ, USA), and formic acid was purchased from Wako (≥99.5%, Wako Pure Chemical Industries, Ltd., Osaka, Japan). Ultrapure water (UW) was prepared using the Puris-Evo UP Water system with Evo-UP Dio VFT and Evo-ROP Dico20 (Mirae ST Co., Ltd., Anyang, Gyeonggi-do, Korea). UW was prepared to have a resistivity of 18.2 MΩ cm^{-1} (Puris, Esse-UP Water system, Mirae St Co., Anyang, Korea). Fetal bovine serum (FBS) and phosphate-buffered saline (PBS) were purchased from HyClone (Logan, UT, USA). Distilled water was filtered through a 0.45-μm membrane filter from ADVANTEC (Tokyo, Japan) before analysis. Dulbecco's modified Eagle's medium (DMEM) was purchased from Lonza (Walkersville, MD, USA). Trypsin/EDTA and penicillin/streptomycin were purchased from Gibco (Grand Island, NY, USA). Anti-phospho-ERK1/2, anti-ERK1/2, anti-phospho-PLCγ1, anti-phospho-STAT3, anti-STAT3, anti-phospho-p38, anti-p38, anti-phospho-Akt, anti-Akt, anti-phospho-JNK, anti-JNK, anti-CDK2, anti-CDK4, anti-cyclin D1, anti-cyclin E1, anti-phospho- Rb, anti-PCNA, anti-p21, anti-p27, and anti-β-actin antibodies were purchased from Cell Signaling Technology Inc. (Beverly, MA, USA). The Cell Counting Kit-8 (CCK-8) was purchased from Dojindo Molecular Technologies (Rockville, MD, USA). In vivo-jetPEI was purchased from Polyplus transfection (Illkirch,

France). Platelet-derived growth factor (PDGF)-BB was obtained from PEPROTECH Co. (Rocky Hill, NJ, USA). All other chemicals were of analytical grade.

Preparation of standard solutions and samples. Each standard stock was prepared by dissolving four standard compounds in 100% methanol at 1 mg/mL. Sample stock solution was extracted 50 mg in 100% methanol by ultrasonic at room temperature for 30 min after filtered through a 0.2 mm syringe membrane filter from Whatman Ltd (Maidstone, UK). All standard and sample solution were stored at 4°C before analysis.

Chromatographic system. Separation was performed in an HPLC system (Hitachi, Tokyo, Japan) comprising a pump, an auto sampler, a column oven and a diode array UV/VIS detector. Chromatograms of detector were registered by the Chromeleone software (version 7) system. The compoenets of *Alpinia officinarum* (AO) were separated on OptimaPak C_{18} column (4.6 × 250 mm, 5 μm, RS tech Co., Daejeon, Korea) at 40°C. The injection volume was 10 μL and the detective wavelength was set at 330 nm. The mobile phase consisting of 0.05% phosphoric acid (A) and acetonitrile (B) was run at a flow rate of 1.0 mL/min. The isocratic elution program was set as condition of 35% B for 60 min [3].

References

1 Ghasemzadeh, A., Jaafar, H. Z. & Rahmat, A. Synthesis of phenolics and flavonoids in ginger (Zingiber officinale Roscoe) and their effects on photosynthesis rate. *Int J Mol Sci* **11**, 4539-4555 (2010).

2 Ghasemzadeh, A., Jaafar, H. Z. & Rahmat, A. Identification and concentration of some flavonoid components in Malaysian young ginger (Zingiber officinale Roscoe) varieties by a high performance liquid chromatography method. *Molecules* **15**, 6231-6243 (2010).

3 Tao , L., Wang, Z.-T., Zhu, E.-Y. & Lu, Y.-H. HPLC analysis of bioactive flavonoids from the rhizome of Alpinia officinarum. *South African Journal of Botany* **72**, 163-166 (2006).

4 Kiuchi, F., Iwakami, S., Shibuya, M., Hanaoka, F. & Sankawa, U. Inhibition of prostaglandin and leukotriene biosynthesis by gingerols and diarylheptanoids. *Chem Pharm Bull (Tokyo)* **40**, 387-391 (1992).

Supplementary Tables

Supplementary Table S1. Calibration curves of five analyses.

Compound	Linear range (µg/ml)	Regression Equation [a]	r^2 [b]
Quercetin	0.1-1000	y = 80185x + 491838	0.9993
Kaempferol	0.1-1000	y = 96004x + 191187	0.9998
Galangin	0.1-1000	y = 69000x + 193364	0.9997
Kaemgferide	0.1-1000	y = 117243x + 238159	0.9998

a) *Y*: peak area, *x*: concentration (µg/mL)

b) Regression coefficient (n=5)

Supplementary Table S2. Calibration curves of five analyses in *Alpinia officinarum* Hance.

Compound	Retention time (time)	Amount (mg/g)	Average (mg/g)	S.D.	R.S.D (%)
Quercetin	ND [a)]	-	-	-	-
Kaempferol	9.82	0.0113	0.0105	0.0013	5.7977
		0.0106			
		0.0119			
Galangin	16.00	1.6777	1.6942	0.0145	0.8558
		1.6998			
		1.7050			
Kaempferide	22.15	0.3331	0.3371	0.0036	1.0734
		0.3380			
		0.3401			

a) ND: not detected

Supplementary Figures

Supplementary Figure S1

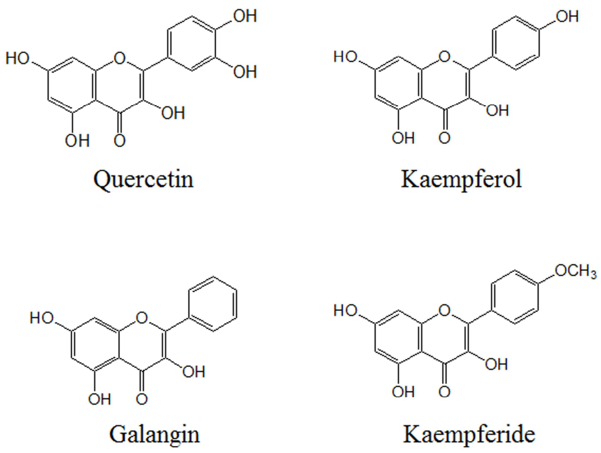

Supplememtrary Figure S1. Chemical structures of quercetin, kaempferol, galangin and kaemferide.

Supplementary Figure S2

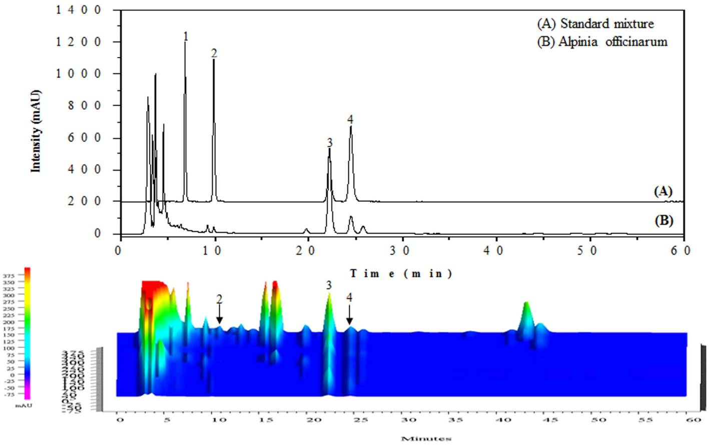

Supplementary Figure S2. Identification analysis of (1) quercetin, (2) kaempferol, (3) galangin, (4) kaempferide by RP-HPLC-DAD (run time: 60 min, mobile phase A: 0.05% phosphoric acid in water, B: Acetonitrile 35% B (0-60 min) injection: 10μl, UV wavelength: 330 nm).

Supplementary Figure S3

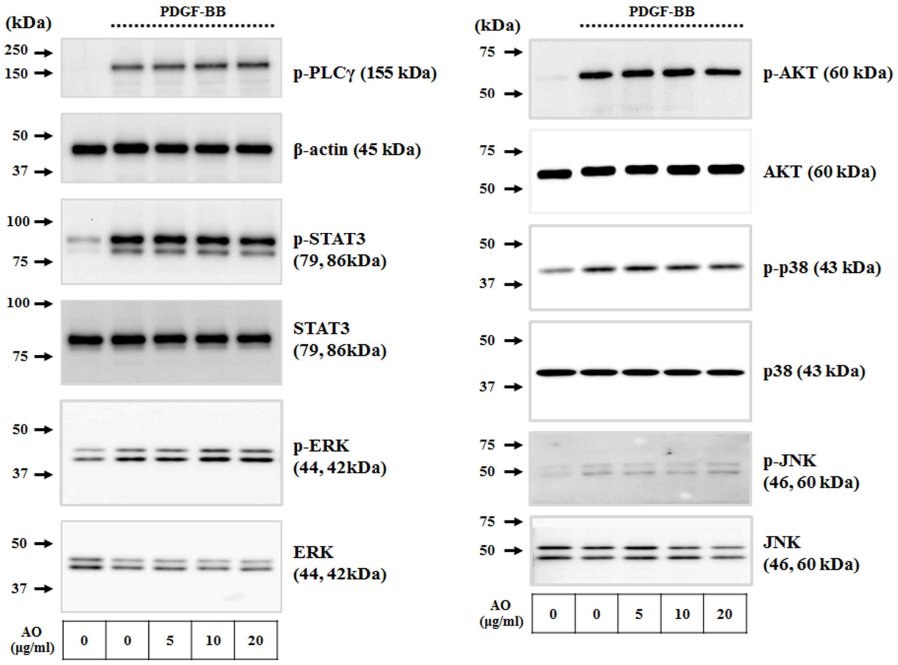

Supplementary Figure S3. Effects of *Alpinia officinarum* Hance (AO) extract on early signaling transduction of vascular smooth muscle cells (VSMCs). This is a full length image of the cropped blots presented in the Figure 1Ca. Protein size marker was purchased from Bio-Rad Co. (Catalog # 161-0374).

Supplementary Figure S4

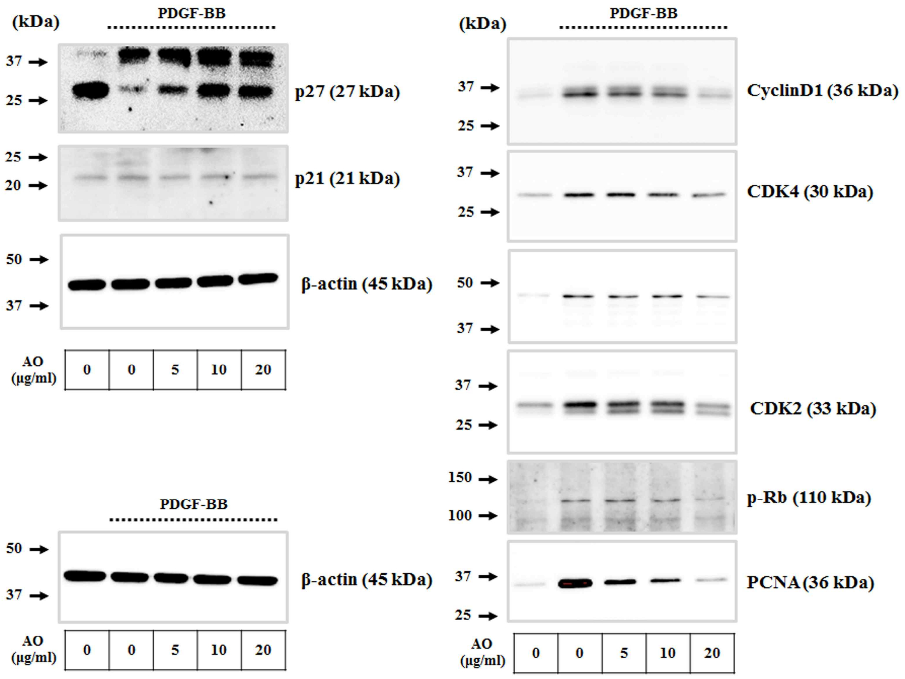

Supplementary Figure S4. Effects of the AO extract on cell cycle regulatory proteins. This is a full length image of the cropped blots presented in the Figure 2C and Da. In addition, effect of AO extract on p21 expression. Protein size marker was purchased from Bio-Rad Co. (Catalog # 161-0374).

Supplementary Figure S5

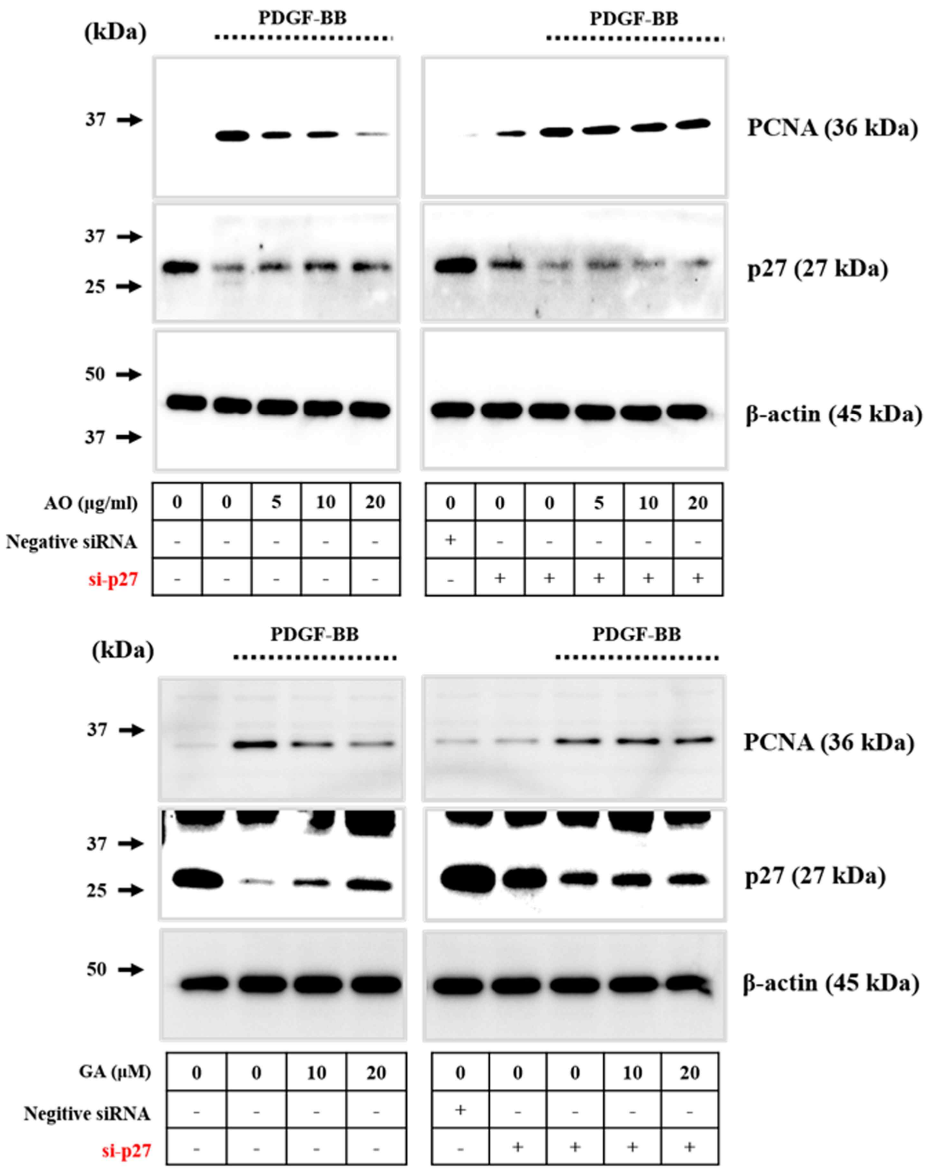

Supplementary Figure S5. Identification of p27 up-regulation as the target of action in the anti-proliferative activity of the AO extract and GA. This is a full length image of the cropped blots presented in the Figure 4Ca and Cc. Protein size marker was purchased from Bio-Rad Co. (Catalog # 161-0374).
